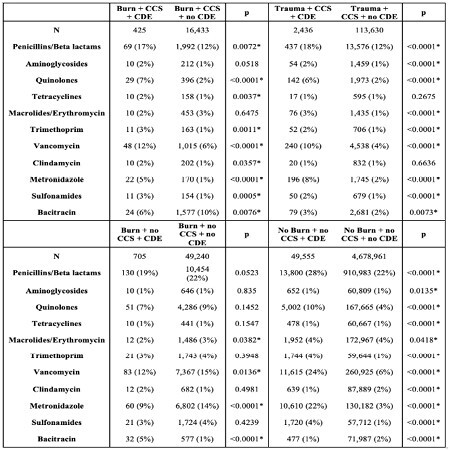# 705 C. difficile Enterocolitis Diagnosis Elevated in Critically Ill Burn Patients Following Antibiotic Treatment

**DOI:** 10.1093/jbcr/irae036.250

**Published:** 2024-04-17

**Authors:** Mathilda Nicot-Cartsonis, Taylor Hallman, Juquan Song, Georgiy Golovko, Steven E Wolf

**Affiliations:** University of Texas Medical Branch at Galveston, Galveston, TX; University of Texas Medical Branch, Blocker Burn Unit, Galveston, TX; University of Texas Medical Branch at Galveston, Galveston, TX; University of Texas Medical Branch, Blocker Burn Unit, Galveston, TX; University of Texas Medical Branch at Galveston, Galveston, TX; University of Texas Medical Branch, Blocker Burn Unit, Galveston, TX; University of Texas Medical Branch at Galveston, Galveston, TX; University of Texas Medical Branch, Blocker Burn Unit, Galveston, TX; University of Texas Medical Branch at Galveston, Galveston, TX; University of Texas Medical Branch, Blocker Burn Unit, Galveston, TX

## Abstract

**Introduction:**

C. difficile colitis (C diff colitis) is identified in up to 500k infections/year in the US, with up to 30,000 people per year dying with the diagnosis. Previous studies established risk factors for this condition including long-term antibiotic use. Burn patients are typically exposed to many antibiotics over long periods of time, and may have lengthy hospitalizations similar to other critically ill patients. Neither of these populations have been studied in terms of C diff colitis, risk and outcomes alike. Our aim is to compare C diff colitis rates among critically ill and non-critically ill burn populations, and to investigate the use of antibiotics prior to diagnosis.

**Methods:**

All analyses were generated with TriNetX platform software (TriNetX, Cambridge, MA) in 2023. We compared incidence of C diff colitis in non-critically ill and critically ill burn populations, and non-burn injury control groups. Cohorts were propensity matched for age at diagnosis, sex, and race/ethnicity. The difference between groups was tested using the log-rank test and quantified with hazard ratios (95% CI), calculated with TriNetX Analytics features. We examined length of hospital stay of cohorts, antibiotic use prior to C diff colitis diagnosis, death within 1 year, and colectomy/toxic megacolon diagnosis.

**Results:**

We found critically ill patients are diagnosed with C diff colitis at similar rates (OR = 1.065, 95% CI = 0.89, 1.276), but non-critically ill burn patients have a higher rate of C diff colitis compared to non-burn injury counterparts (OR = 1.713, 95% CI = 1.471, 1.995). Antibiotics use up to 2 weeks prior to C diff colitis diagnosis was higher in C diff colitis patients in all four cohorts. Specifically for critically ill burn C diff colitis patients, penicillin (p=0.0072), quinolones (p< 0.0001), tetracyclines (p=0.0037), trimethoprim (p=0.0011), vancomycin (p< 0.0001), clindamycin (p< 0.0357), metronidazole (p< 0.0001), and sulfonamides (p=0.0005) were all given more frequently as compared to counterparts without C diff colitis.

**Conclusions:**

Critically ill burn patients are at an increased risk of C diff colitis compared to non-critically ill burn, as are critically ill non-burn patients. C diff colitis remains significantly associated with antibiotic use, and in the setting of critical illness and burn, is a notable risk factor.

**Applicability of Research to Practice:**

C diff colitis remains a threat to hospitalized and burn patients, for the critically and non-critically ill, and antibiotics treatment could be more aware of the worse consequence.